# TB yields from expanded contact tracing investigations

**DOI:** 10.5588/pha.24.0052

**Published:** 2025-03-01

**Authors:** A. Madan, A.A. Malik, M.B. Brooks

**Affiliations:** ^1^ Boston University School of Public Health, Boston, MA, USA;; ^2^ University of Texas Southwest, Dallas, TX, USA;; ^3^ Interactive Research & Development Global, Singapore.

**Keywords:** pediatric TB, smear-negative TB, extrapulmonary TB

## Abstract

**BACKGROUND:**

TB poses a significant global health challenge due to a substantial case-detection gap. Traditional contact tracing primarily targets contacts of bacteriologically confirmed pulmonary TB index patients, often excluding pediatric, clinically diagnosed, and extrapulmonary cases. This review assessed the potential of expanding contact tracing to these frequently overlooked subgroups.

**METHODS:**

We conducted a focused, targeted literature review by searching PubMed, Web of Science, Google Scholar and Lens.org using identified keywords. A title and abstract review was conducted using predefined inclusion/exclusion criteria.

**RESULTS:**

We identified 13 relevant studies reporting contact tracing yields from these index patient groups. Contact tracing of pediatric, clinically diagnosed, and extrapulmonary TB index patients yielded up to 8.1%, 3.0%, and 2.1% for active disease and up to 17.9%, 12.6%, and 11.1% for TB infection, respectively.

**CONCLUSION:**

Findings suggest that expanding contact tracing for these typically excluded index patients can improve case detection. By refining contact tracing protocols and adopting more inclusive strategies, TB programs can enhance case detection rates and improve overall disease control efforts, aligning with global goals for TB elimination.

TB remains a critical global health issue with 1.25 million deaths and an estimated 10.8 million new cases in 2023.^1^ However, only 8.2 million were notified to health systems, leaving a 2.7 million case-detection gap,^1^ which perpetuates transmission, morbidity, and mortality.

In 2015, the WHO launched the End TB Strategy to achieve a TB-free world, targeting ‘zero deaths, disease, and suffering due to TB.’^2^ Key goals include 90% contact investigation coverage for individuals with bacteriologically confirmed TB.^3^ And 90% treatment coverage of estimated TB cases by 2025.^4^ While thorough contact tracing can support these targets, achieving treatment coverage requires identifying individuals at high risk of TB who are overlooked in routine investigations.

Contact tracing systematically identifies individuals exposed to TB patients, with close and household contacts having a 3.6% pooled prevalence of TB.^5^ It typically focuses on contacts of adults with bacteriologically confirmed pulmonary TB, multidrug-resistant TB, individuals living with HIV, and children under 5 years old,^5^ efficiently allocating resources but excluding many individuals with TB—such as children, those without bacteriologic confirmation, and those with extrapulmonary TB (EPTB). Expanding contact tracing to these groups could help close the case-detection gap and meet treatment coverage targets. This review examines whether conducting contact tracing efforts for index patients that are typically excluded from contact tracing guidelines, including children (5–15 years of age), individuals without bacteriologic confirmation of disease, and individuals diagnosed with EPTB, could increase TB detection.

## METHODS

We conducted a focused, targeted literature review, which aims to be informative rather than all-encompassing. As such, a formal but restricted search strategy was devised. First, we identified relevant keywords related to the research question and explored these controlled terms across multiple databases, including PubMed, Web of Science, Google Scholar, and Lens.org. Specific search terms were meant to identify relevant studies, though not to be fully comprehensive. Terms included were: ‘Tuberculosis Pulmonary’, ‘Tuberculosis Extrapulmonary’, ‘smear-negative TB’, ‘clinically diagnosed TB’, ‘contact tracing’, ‘contact tracing in extrapulmonary TB’, ‘extrapulmonary TB contact’, ‘nonpulmonary TB’, ‘non-pulmonary TB contact tracing’, ‘active case finding tuberculosis’, ‘case finding tuberculosis’, ‘pediatric TB,’ and further variations of ‘contact tracing in TB’. Medical Subject Headings and specific Boolean search queries were employed. We conducted manual citation chaining on reference lists from identified appropriate systematic reviews.

As is standard with a focused, targeted literature review, we limited the review to only one reviewer who only reviewed the titles and abstracts using defined inclusion and exclusion criteria. Inclusion criteria involved research studies comprising index TB patients who are typically not focused upon during routine contact tracing efforts—individuals with EPTB, those without bacteriologic confirmation of TB disease, and children with TB (5–14 years old, though studies including children <15 years old or where ages of children were not disaggregated were also eligible). We excluded studies that did not include TB contact tracing, that only included contact tracing of adult patients with pulmonary, bacteriologically confirmed TB and those that did not report the TB yield of the contact tracing efforts. We did not geographically limit our search, although we only included original research studies published in English and conducted after 1990 through May 2024. We considered all research methodologies, including retrospective and prospective studies. Full-text articles were obtained for those that passed the initial title and abstract check to determine their final relevance and eligibility.

Once the final set of articles was selected based on our criteria, we extracted the following data: author name, study date, study design, study site and its WHO regional categorization,^6^ definition of the index patient, type of index patient group contact tracing was done for, number of index patients per group type, definition of contacts, number of contacts identified by index patient type, TB disease and TB infection yields by index patient type, and whether contacts outside of the household were included in the studies or not.

To evaluate the quality and reliability of studies, we observed key parameters like study design, sample size, and screening tests used (tuberculin skin test [TST] or interferon-gamma release assay [IGRA] and their implementation protocols). To ensure consistency in diagnostic and screening procedures, we examined the diagnostic criteria researchers used to differentiate between clinically diagnosed and bacteriologically confirmed diseases. Additionally, we scrutinized the criteria for defining a positive result from the screening tests. To assess the quality of evidence, we utilized the Oxford Centre for Evidence-Based Medicine (OCEBM) classification system, which categorizes studies into five levels based on methodological rigour and potential for bias, with Level 1 representing the highest quality of evidence and Level 5 the lowest.^7^ For this review, we treated all studies as cross-sectional by analyzing the prevalence of TB in the population as if it were captured at a specific point in time, regardless of the original study design. This method facilitated direct comparisons between studies by concentrating on prevalence data, which is crucial for evaluating contact tracing outcomes. We employed this approach to ensure that our conclusions were drawn based on a uniform and unbiased assessment of available data.

### Ethics approval

This study involved a review of previously published studies that are publicly available. As no individual data were assessed, ethics approval was not required.

## RESULTS

After reviewing 16 full-text articles of those eligible after title/abstract review, three were excluded for not reporting TB screening yields, and 13 studies were included from diverse settings. Nine were from the European Region,^8–16^ 2 each from the African Region^17,18^ and Eastern Mediterranean Regions,^19,20^ spanning 1990–2020 (Table 1).

**TABLE 1.** Detailed descriptions of studies included in the final review detailing TB yield from varying index patient groups.

Author name, reference	Study dates	WHO Region	Study site	Study design	Definition of index patients	Index patient group	Definition of contacts	Number of contacts identified	TB disease yield	LTBI yield
Sloot^16^	2008–2011	EUR	Amsterdam, The Netherlands	RCS	SS+ and SS– PTB patients reported to the Public Health Services	SS– (*n* = 129)	Contacts were identified based on their exposure to PTB index patients, and classified into first-, second-, or third-circle contacts according to the duration and frequency of exposure during the infectious period	444 (plus 364 casual contacts that were excluded)	Not reported for index patient group of interest	9 (4%)
Cavany^8^	2012–2015	EUR	London, UK	RCS	No definition provided	EPTB (*n* = 2,943) SS– (*n* = 1,095) Pediatric <15 years (*n* = 124)	Close contacts of index cases with TB	EPTB (*n* = 7,264) SS– (*n* = 3,421) Pediatric <15 years (*n* = 491)	EPTB (*n* = 51, 0.7%) SS– (*n* = 45, 1.3%) Pediatric <15 years (*n* = 27, 5.5%)	Not reported for index patient group of interest
Saunders^9^	1990–2010	EUR	Birmingham, UK	RCS	Patients with PTB, defined as involvement of the lung parenchyma, mediastinal lymph nodes or pleaura, or EPTB	EPTB (*n* = 2,309) SS– *(n* = 3,514*)*	A contact with >8 h of exposure per day was defined as a close contact and invited to be included in the first ‘ring’ of contacts. If >10% of the first ring of contacts screened had a positive tuberculin skin test, the screening team invited a second ring of contacts to participate (usually friends, workplace colleagues and more distant relatives)	EPTB (*n =* 9.875*)* SS– (*n =* 15,335*)* (includes only those who had a screening outcome available)	EPTB (*n* = 57, 0.6%) SS– (*n* = 181, 1.2%)	EPTB (*n* = 165, 1.7%) SS– (*n* = 440, 2.9%)
Underwood^10^	1997–1999	EUR	UK	RCS	Residents notified as having TB over a 3-year period with either PTB (SS+ or SS–) or non-pulmonary disease	EPTB (*n* = 83) SS– (*n* = 78)	Close contacts (typically household contacts) of patients with PTB (SS+ or SS–); casual contacts of patients with SS+ disease only if they are children or if the index patient is highly infectious (i.e., has infected more than 10% of close contacts); contacts of patients with non-pulmonary disease only if the index case is likely to have been infected recently (for example, a child)	EPTB (*n* = 227) SS– (*n* = 156)	EPTB (*n* = 2, 0.9%) SS– (*n* = 3, 1.9%)	Provided prophylaxis after TB disease screening: EPTB (*n* = 1. 12, 5.3%) SS– (*n* = 2. 9, 5.8%)
Mandal^11^	2008–2010	EUR	Edinburgh, UK	PCS	Patients with TB, including those with pulmonary and non-pulmonary involvement	EPTB (*n* = 115) SS– (*n* = 72)	Household contacts: Those who share a bedroom, kitchen, bathroom or sitting room with the index case. Casual contacts: Workplace contacts	EPTB (*n* = 233) SS– (*n* = 167) (includes only those who had a screening outcome available)	EPTB (*n* = 5, 2.1%) SS– (*n* = 5, 3.0%)	EPTB (*n* = 25, 10.7%) SS– (*n* = 21, 12.6%)
Andrews^12^	2011–2020	EUR	Birmingham, UK	RCS	TB cases entered into the database at University Hospitals Birmingham NHS Foundation Trust, as well as Enhanced TB Surveillance	EPTB (*n* = 1,189) SS– (*n* = 1,032)	No definition provided	EPTB (*n* = 4,884) SS– (*n* = 11,444)	EPTB (*n* = 13, 0.3%) Not reported for SS–	EPTB (*n* = 244, 5.0%) Not reported for SS–
van de Berg^13^	2011–2016	EUR	The Netherlands	RCS	No definition was provided, but included TB patients registered in the Netherlands Tuberculosis Register with a contact investigation initiated	EPTB (*n* = 1,053) SS– (*n* = 956) Pediatric <15 years (*n* = 105)	Contacts are prioritized for testing in concentric circles around the index patient, inclusive of close, casual, and community contacts	Casual + community contacts EPTB (*n* = 897) SS– (*n* = 5,806) Pediatric <15 years (*n* = 722)	Casual + community contacts: EPTB (*n* = 0, 0%) SS– (*n* = 9, 0.2%) Pediatric <15 years (*n* = 2, 0.3%)	Casual + community contacts: EPTB (*n* = 40, 6.0%) SS– (*n* = 179, 4.0%) Pediatric <15 years (*n* = 39, 6.2%)
								Close contacts: EPTB (*n* = 3,994) SS– (*n* = 5,277) Pediatric <15 years (*n* = 895)	Close contacts: EPTB (*n* = 28, 0.8%) SS– (*n* = 23, 0.5%) Pediatric <15 years (*n* = 23, 2.7%)	Close contacts: EPTB (*n* = 332, 11.1%) SS– (*n* = 393, 9.3%) Pediatric <15 years (*n* = 143, 17.9%)
Wingfield^14^	2012–2016	EUR	England, UK	RCS	Adult (≥18 years old) with microbiologically or clinically confirmed EPTB with ≥1 household contact and <25 contacts identified	EPTB (*n* = 1,026)	No definition provided.	3,652	0.44% (95% CI 0.2–0.6)	3.6% (95% CI 2.7–4.5)
Migliore^15^	2002–2008	EUR	Turin, Italy	RCS	Suspected of confirmed PTB case	SS– (not reported)	Anyone having shared air with an active TB case; inclusive of household members, close contacts, and regular contacts, defined according to the stone-in-the-pond method	Household contacts: *n* = 1,305 S–C+ (*n* = 613) S–C+ (*n* = 692)	Not reported for patient groups of interest	Contacts of sputum S-C+ index patients had a statistically significantly higher risk of TB infection than did the contacts of sputum S–C– index patients (OR 2.93, 95% CI 1.34–6.41)
Kisamba^17^	2018–2020	AFR	Uganda	RCS	Index patients with TB reported to the Uganda National TB and Leprosy Programme	No bacteriological confirmation and EPTB combined (not reported) Pediatric <15 years (not reported)	Not provided	No bacteriological confirmation and EPTB combined (*n* = 4,999) Pediatric <15 years (*n* = 3,919)	No bacteriological confirmation and EPTB combined (*n* = 100, 2.0%) Pediatric <15 years (*n* = 58, 1.5%)	—
Puryear^18^	2009–2011	AFR	Gaborone, Botswana	PCS	Children aged 0–13 years with TB diagnosed by a paediatrician	Pediatric ≤13 years (*n* = 163)	Any person, regardless of age, who had slept at least one night under the same roof as the index case within 6 months prior to the index patient initiating anti-TB treatment	548	12 (2.2%)	—
Jaswal^19^	2018–2019	EMR	Karachi, Pakistan	PCS	Index patients with clinically diagnosed PTB or index patients with EPTB who are at least 5 years old	EPTB (*n* = 1,132) SS– (*n* = 1,833)	A person sharing the same kitchen under the same roof with an average exposure to a TB patient of 6-8 hours a day for 5-7 days a week during the last 3 months	EPTB (*n* = 3,074) SS– (*n* = 6,965)	EPTB (n = 50, 1.6%) SS– (*n* = 147, 2.1%)	—
Malik^20^	2014–2016	EMR	Sindh, Pakistan	PCS	Children under 15 years diagnosed with TB	Pediatric <15 years (not reported)	Household contacts (specific definition not provided)	2,679	217 (8.1%)	—

LTBI = latent TB infection; EUR = WHO European Region; RCS = randomized controlled study; S–C– = sputum smear-negative and culture negative; S–C+ = sputum smear-positive and culture-positive; SS+ = sputum smear-positive; SS– = sputum smear-negative; PTB = pulmonary TB; EPTB = extrapulmonary TB; AFR = WHO Africa Region; EMR = WHO Eastern Meditteranean Region.

### Definitions of contacts

Several studies included household contacts, while some included workplace contacts, friends, and community members. Mandal et al. included workplace contacts as casual contacts,^11^ while Sloot et al. classified contacts into first, second, or third circles based on exposure intensity,^16^ per the standard approach in the Netherlands.^21^ Migliore et al. defined three categories of contacts: household, regular (e.g., friends and colleagues), and occasional.^15^ Van de Berg et al. also included casual and community contacts.^13^ Saunders et al. included contacts outside the household and expanded their tracing efforts to the second ring of contacts when necessary.^9^ TB disease and TB infection yields were not consistently reported for non-household contacts.

### Index patients with EPTB

Seven European,^8–14^ one African,^17^ and one Eastern Mediterranean^19^ studies reported TB yields of contact tracing efforts for index patients with EPTB. TB disease yields among contacts ranged from 0.3% to 2.1%.^8–14,17,19^ Four studies reported a TB infection yield, ranging from 1.7% to 11.1%.^9–14^

### Index patients lacking bacteriologic confirmation of disease

Of 10 studies of contact tracing efforts for index patients with sputum smear-negative TB,^8^ were from Europe,^8–13,15,16^ one in Africa,^17^ and one in the Eastern Mediterranean.^19^ Seven reported the contact TB disease yield, ranging from 0.2% to 3.0%.^8–11,13,17,19^ Five reported a TB infection yield ranging from 2.9% to 12.6%.^9–11,13,16^ A sixth study reported that contacts of sputum smear-negative, culture-positive index patients had a statistically significantly higher risk of TB infection than did contacts of sputum smear-negative, culture-negative index patients (odds ratio [OR] 2.93, 95% confidence interval [CI] 1.34–6.41).^15^

### Pediatric index patients

Contact tracing for pediatric TB index patients was reported in 5 studies: 2 from Europe,^8,13^ 2 from Africa,^17,18^ and 1 from the Eastern Mediterranean Region.^20^ Cavany et al. included 124 index patients <15 years old from the United Kingdom and found 491 contacts, of which 27 (5.5%) were diagnosed with TB.^8^ Van de Berg et al.’s study, conducted in the Netherlands identified 105 index patients <15 years old. In total, 895 close contacts were identified, with 23 (2.7%) having TB disease and 143 (17.9%) having TB infection. An additional 722 casual and community contacts were identified, yielding 2 (0.3%) with TB disease and 39 (6.2%) with TB infection.^13^ Kisamba et al. identified 3,919 contacts of an unknown number of index patients <15 years old in Uganda. Fifty-eight (1.5%) contacts were diagnosed with TB disease.^17^ Puryear et al. conducted contact tracing of 163 pediatric index TB patients <13 years old in Botswana; of 548 identified contacts, 12 (2.2%) had TB disease.^18^ Malik et al. in Pakistan found 217 cases from 2,679 household contacts of index patients aged <15 years and reported a yield of 8.1%.^20^ These findings illustrate the variability in disease yields among pediatric contacts and emphasize the need for comprehensive contact tracing approaches tailored for children.

All studies were graded as having an evidence level of 2 per the OCEBM grading criteria (Table 2). This signifies reliable and consistent application of screening methods and diagnostic criteria across all studies and further enhances the reliability of the findings.

**TABLE 2.** Evidence grade of study per the Oxford Centre for Evidence-Based Medicine classification system*

#	Title	Author	Study dates	Evidence level	Design
1	Yield of tuberculosis contact investigations in Amsterdam: opportunities for improvement	Sloot^16^	2008–2011	2b	Treated as cross-sectional. Screening methods (TST/IGRA, CXR), diagnostic criteria and preventive treatment are uniformly applied
2	An evaluation of tuberculosis contact investigations against national standards	Cavany^8^	2012–2015	2b	Treated as cross-sectional. Screening methods (TST/IGRA, CXR) and diagnostic criteria are consistently applied
3	Predictors of contact tracing completion and outcomes in tuberculosis: a 21-year retrospective cohort study	Saunders^9^	1990–2010	2b	Treated as cross-sectional. Definitions (EPTB, PTB, screening completion, positive outcome), stratification, smear testing, were consistently applied
4	Contact tracing and population screening for tuberculosis–who should be assessed?	Underwood^10^	1997–1999	2b	Treated as cross-sectional. Categorization of index cases (S+ PTB, S– PTB, EPTB), definition of outcomes (active TB cases detected, chemoprophylaxis given) and use of guidelines for decision-making on chemoprophylaxis were consistently applied and had clear definitions
5	Contact tracing in pulmonary and non-pulmonary tuberculosis	Mandal^11^	2008–2010	2b	Treated as cross-sectional. Study has defined diagnosis of active TB (according to national guidelines), of latent TB (based on history), TST, and IGRA and also has consistent definitions of household and casual contacts, positive screening episodes. It also mentions using national guidelines for diagnosis and treatment
6	Outcomes of TB contact tracing and predictors of success: a 10-year retrospective cohort analysis in Birmingham, UK	Andrews^12^	2011–2020	2b	Treated as cross-sectional. Clearly defined diagnostic criteria, TB classifications (PTB or EPTB, LTBI), and screening procedures (smear-testing, IGRA). These standards have been applied consistently across the entire study period.
7	Tuberculosis contact investigation following the stone-in-the-pond principle in the Netherlands – Did adjusted guidelines improve efficiency?	van de Berg^13^	2011–2016	2b	Treated as cross-sectional, originally retrospective. Study has clear definitions for priority groups of contacts, standardized procedures based on national guidelines
8	High prevalence of TB disease in contacts of adults with extrapulmonary TB	Wingfield^14^	2012–2016	2b/3	Treated as cross-sectional. Has consistently applied screening procedures but does not have randomization and a control group
9	Outcomes of a tuberculosis contact investigation programme in Italy	Migliore^15^	2002–2008	2b	Treated as cross-sectional, originally population-based retrospective study. Study has consistent data sources and clear eligibility criteria
10	Tuberculosis yield among contacts of non-pulmonary bacteriologically confirmed index TB patients in the urban setting of central Uganda	Kisamba^17^	2018–2020	2b	Treated as cross-sectional, originally retrospective cohort. Has clear definitions of cases, had standardized screening process for TB contacts, and consistent definition of TB diagnosis among contacts
11	Yield of contact tracing from pediatric tuberculosis index cases in Gaborone, Botswana	Puryear^18^	2009–2011	2b	Treated as cross-sectional, originally retrospective cohort. Study has clear definition of cases, standardized processes, screening outcomes and new TB cases in contacts
12	TB disease yield from household contact screening of tuberculosis index patients; a cohort study from Karachi, Pakistan	Jaswal^19^	2018–2019	2b	Treated as cross-sectional, originally prospective cohort. Study has clear and consistent definition of cases and contacts and standardized screening process
13	High yields from contact investigation of child index TB	Malik^20^	2014–2016	2b	Treated as cross-sectional. Study has standardized screening procedures along with clear and consistent diagnostic criteria

* The Oxford Centre for Evidence-Based Medicine (OCEBM) classification system categorizes studies into five levels based on methodological rigour and potential for bias, with Level 1 representing the highest quality of evidence and Level 5 the lowest.

CXR= chest X-ray; TST= tuberculin skin test; IGRA = interferon gamma release assay; EPTB = extrapulmonary TB; PTB = pulmonary TB; S– = smear-negative; S+= smear-positive; LTBI = latent TB infection.

## DISCUSSION

Our review emphasizes the need to include traditionally overlooked index-patient groups, such as EPTB and smear-negative/clinically diagnosed patients, in contact tracing. EPTB contact tracing, often deprioritized due to perceived lower infectiousness, showed a TB disease yield of approximately 2% in reviewed studies. Expanding contact tracing to these groups could identify individuals with undiagnosed TB and reduce transmission. With a global TB incidence of 133 per 100,000 in 2022,^4^ early detection and comprehensive management remain critical. In Karachi, Pakistan, household contacts of patients with clinically diagnosed TB and EPTB TB were as likely to be diagnosed with TB as those of patients with bacteriologically confirmed TB.^19^ Our review also found that tracing contacts of pediatric TB patients can result in 1.5–5.5% of the contacts being diagnosed with TB. This may seem counterintuitive to the established wisdom that children are unlikely to transmit the disease. However, often, the child might be the first person to be diagnosed with the disease, while there might be an undiagnosed adult in the household who may be spreading the disease. Multiple people in the household may have come in contact with someone with TB, and the child may be the first to be diagnosed. Studies in Pakistan by Malik et al. showed that screening the household with a child TB patient may result in more children being diagnosed with TB.^19,20^ Traditional focus on smear-positive patients has often overlooked significant yields that contact tracing in other groups has produced. This stresses the need for further research into improving contact tracing strategies to make them more adaptable across different TB subgroups and healthcare settings. Addressing these needs, alongside investing in healthcare infrastructure and support systems, can substantially improve TB treatment outcomes and reduce TB-related mortality. Narrowing the gaps in TB contact tracing across different patient subgroups will require targeted interventions like expanding contact tracing programs and improving diagnostic capacities. This targeted focused literature review aimed to identify the usefulness of expanding the definition for which index patient contact tracing efforts should be employed. To that end, the targeted, focused literature review is not meant to be comprehensive or all-encompassing of every study in this field; instead, it serves as a rapid demonstration that expanding these efforts to index patients typically not included in the guidelines can increase the yield of TB detected, ultimately aiding in closing the case-detection gap globally. Nonetheless, we robustly assessed the studies’ methodological quality to ensure valid conclusions could be drawn.

## CONCLUSION

Our review stresses the importance of expanding contact tracing efforts in TB control to include often-overlooked subgroups, such as those without bacteriologic confirmation and patients with extrapulmonary TB. Evidence from both high- and low-burden settings shows that contact tracing within these groups leads to significant clinical outcomes, emphasizing its potential to reduce global TB burden. TB programs can enhance case detection rates and improve overall disease control by refining contact tracing protocols and adopting more inclusive strategies. Future research should prioritize optimizing contact tracing models for non-traditional index cases to bridge detection gaps and ensure more comprehensive TB control efforts worldwide. Further, more thorough, systematic reviews and meta-analyses can be employed to better understand the pooled TB yields for each index-patient group, including the associated diagnosis methods, contact investigation strategies, and associated characteristics.
